# Health-related quality of life (HRQoL) assessment with once- and twice-daily darunavir/ritonavir (DRV/r) in the ODIN trial

**DOI:** 10.1186/1758-2652-13-S4-P26

**Published:** 2010-11-08

**Authors:** S Spinosa-Guzman, T Van de Casteele, G De La Rosa, P De Doncker

**Affiliations:** 1 Tibotec BVBA, Beerse, Belgium; 2 Tibotec Therapeutics, Titusville, NJ, USA

## Background

The open-label, Phase III, ODIN trial randomised treatment-experienced HIV-1-infected patients with no DRV resistance-associated mutations (RAMs) to receive DRV/r 800/100mg qd or DRV/r 600/100mg bid, plus an optimised background regimen (≥2 NRTIs). Non-inferiority in the primary endpoint of virological response at Week 48 was demonstrated with DRV/r qd versus bid dosing: 72.1% vs 70.9% of patients, respectively, achieved HIV-1 RNA <50 copies/mL (95% CI: -6.1, 8.5; p<0.001; ITT-TLOVR). The current analysis explores patient-reported HRQoL.

## Methods

Treatment-experienced patients with no DRV RAMs at screening and HIV-1 RNA >1,000 copies/mL were randomised. Patient-reported HRQoL was measured with the Functional Assessment of HIV-infection (FAHI) questionnaire at baseline and at Weeks 4, 12, 24 and 48 (or withdrawal visit). FAHI score at Week 48 was modelled by means of an ANCOVA, and the evolution of the FAHI score over time by means of a longitudinal mixed model, each with treatment as a factor and CD4 and baseline HIV-1 RNA as a regressor. FAHI response was defined as the proportion of patients with a clinically meaningful difference (relative increase of 10%) in total FAHI imputed score versus baseline.

## Results

HRQoL data were available for 262/294 DRV/r qd and 268/296 DRV/r bid patients. The baseline total FAHI imputed score was relatively high (124.1 and 121.2 for DRV/r qd and bid, respectively), leaving limited room for improvement. Mean (SE) increase in total FAHI score from baseline (ITT-LOCF) at Week 48 was comparable with DRV/r qd and bid dosing (table 1). A mean increase in total FAHI score from baseline was observed in both treatment groups at all timepoints. No relevant between-group differences were noted either by ANCOVA (p=0.761) or longitudinal mixed model (p=0.995). There were no relevant differences between arms at any time in the proportion of FAHI responders (p=0.957).

**Table 1:** 

	DRV/r 800/100mg qd (n=262)	DRV/r 600/100mg bid (n=268)
Mean (SE) change in total FAHI score from baseline (ITT-LOCF)		

Baseline value	124.1 (1.78)	121.2 (1.73)

Week 4	0.4 (1.10)	1.3 (1.08)

Week 12	3.2 (1.18)	1.7 (1.28)

Week 24	2.8 (1.27)	2.5 (1.26)

Week 48	2.7 (1.36)	3.1 (1.40)

FAHI responders (LOCF) at Week 48, %	27.5	29.5

## Conclusions

DRV/r qd and bid dosing was comparable with respect to the increase in mean total FAHI score from baseline at Week 48 and in the proportion of patients achieving a clinically meaningful difference in total FAHI score at Week 48.

